# Two‐scale topology optimization in computational material design: An integrated approach

**DOI:** 10.1002/nme.5742

**Published:** 2018-01-10

**Authors:** A. Ferrer, J.C. Cante, J.A. Hernández, J. Oliver

**Affiliations:** ^1^ Centre Internacional de Mètodes Numèrics en Enginyeria Universitat Politècnica de Catalunya, Campus Nord Barcelona Spain; ^2^ Escola Superior d'Enginyeries Industrial Aeroespacial i Audiovisual de Terrassa, Universitat Politècnica de Catalunya, Campus de Terrassa Terrassa Spain; ^3^ Escuela Técnica Superior de Ingenieros de Caminos, Canales y Puertos de Barcelona Universitat Politècnica de Catalunya, Campus Nord Barcelona Spain

**Keywords:** level sets, material design, multiscale, topology optimization

## Abstract

In this work, a new strategy for solving multiscale topology optimization problems is presented. An alternate direction algorithm and a precomputed offline microstructure database (Computational Vademecum) are used to efficiently solve the problem. In addition, the influence of considering manufacturable constraints is examined. Then, the strategy is extended to solve the coupled problem of designing both the macroscopic and microscopic topologies. Full details of the algorithms and numerical examples to validate the methodology are provided.

## 1 Introduction

Topology optimization has gained considerable popularity in the recent years as a mathematical tool for addressing engineering design problems. The popular SIMP (solid isotropic material with penalization) method,^1^ the shape optimization method by means of a level set function,^2^ the topological derivative,^3^ and the heuristic BESO (bidirectional evolutionary structural optimization) method^4^ are arguably the most used strategies to solve topology optimization problems. In this work, we adopt the topological derivative approach for the following reasons: (i) mathematical consistency; (ii) it is free of heuristic parameters; (iii) it is able to nucleate new holes; and (iv) the optimal topologies are, in general, free of grays.

Although topology optimization has been usually applied to design the structure of a given domain, it can be also employed for material design problems.^1^ The framework provided by multiscale and computational homogenization techniques, such as the asymptotic expansion^5^ or the variational multiscale method,^6^ has allowed the design of materials by using topology optimization in the microscale.

In the specific case of topological derivative, considerable success has been achieved in solving both the macroscopic topology optimization problem(3, 7) and its microscopic counterpart.^8^ However, the design of both scales has been tackled in an uncoupled way. The coupled two‐scale topology optimization problem has been addressed in the related literature (see other works(9, 10, 11, 12)) but using other techniques such as the SIMP or BESO.

This work draws on our recent work,^13^ which tackles the problem of deciding which kind of materials, ie, microscopic topologies, are appropriate to optimize some certain cost function at the macroscale. Hereafter, we shall refer to this problem as the *point‐to‐point* (P2P) material design problem. In order to reduce the resulting exorbitant computational cost, we propose in our recent work^13^ to precompute the microstructures by solving all the possible microscopic topology optimization problems once and, then, save the solutions in a material catalog (database), which, borrowing the term from the work of Chinesta et al,^14^ will be termed *Computational Vademecum*. Then, when solving the multiscale problem, only a straightforward selection of the microstructure entry in the *Computational Vademecum* is required.

The first goal of this work is to modify the approach in our recent work^13^ to account for *manufacture constraints*. The interest of providing not only optimal but also manufacturable topologies is notorious in the works found in the literature. For instance, Peralta and Fachinotti^15^ proposed a novel strategy to deal with manufacturing constraints choosing the material at a given point from a set of predefined metamaterials. Schury et al^11^ tackled manufacture constraints by adding conditions to the spatial variation of the volume fraction. Alternatively, the approach proposed by Kato et al^10^ imposes the same microstructure over all the domain. In contrast, in this work, we advocate to divide the macroscopic domain in components and provide the optimal solution considering the same microstructure for every component. We shall refer to this approach as the *component‐based* (CB) material design problem. We provide the full details of the proposed algorithm and some numerical examples to validate the methodology. In addition, we study the CB *approach consistency*, ie, how the CB material design problem converges to the P2P one, when the defined components tend to single finite elements in the macrostructure.

Another contribution of this work is an approach for simultaneous designing, in a coupled way, of the macroscopic and microscopic topologies. We combine the Slerp (spherical linear interpolation) algorithm proposed in the work of Amstutz and Andrä^3^ for solving the macroscopic topology with the P2P and CB algorithms proposed to solve the material design problem. These strategies lead to the P2P and the CB multiscale topology optimization problem. We also provide full details of the algorithm and numerical examples.

In Section 2, we describe the topological derivative approach for solving topology optimization problems in the macroscale and in the microscale. In Section 3, the P2P approach is briefly described and the CB approach is introduced and formulated in detail. The strategy and algorithm to solve the problem are presented and illustrated with some numerical examples. In Section 4, the resulting two‐scale topology optimization problem and solution algorithm are then presented, and numerical results for the two‐scale topology optimization are provided. Finally, Section 5 summarizes the results of this work and draws some concluding remarks.

## 2 Topological Derivative for Multiscale Topology Optimization

The main virtue of the topological derivative is that it provides the sensitivity of the cost function when an infinitesimal topological perturbation, normally circular, is created. The basis for derivation of the topological derivative was established in the work of Sokolowski and Zochowski^16^ using the results for shape sensitivity, consolidating the theory later in the work of Novotny and Sokolowski.^17^ The works of Novotny et al^18^ and Amstutz^19^ deserve also a special attention. On the other hand, one of the main drawbacks of topological derivative techniques lies on the difficulty of obtaining such topological derivative, which in some cases is technical and tedious. In fact, up to now, for some specific cost functions, topological derivatives are still missing. However, relevant advances have been achieved in the last years about this issue.

In their seminal work,^3^ Amstutz and Andrä advocated the use of the topological derivative as a descent direction in a level‐set algorithm. Slerp, as this algorithm is known in other communities like computer graphics, provides an efficient strategy for solving the topology optimization problem. On one hand, a clear boundary of the topology is found once the algorithm converges, and on the other hand, and in contrast with shape optimization based algorithms, nucleation of holes appears naturally. Certainly, the topological derivative appears a useful tool for solving topological optimization problems, getting rid of heuristic parameters.

### 2.1 Topological derivative

**Figure 1:**
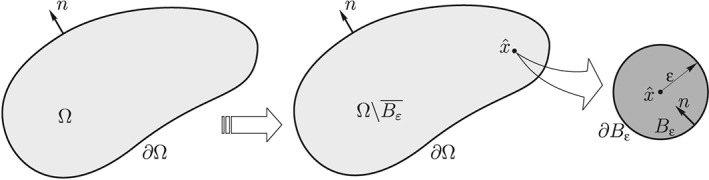
The topological derivative concept

Let us consider an unperturbed domain $\Omega \subset R^{2}$ in which the compliance function is defined as

(1) $$J(\Omega )=\frac{1}{2}\int_{\Omega }\sigma :C^{-1}:\sigma \;d\Omega ,$$

where C stands for the fourth‐order elasticity tensor and σ for the stresses in an elastic equilibrium problem. Then, we consider a perturbed domain by inserting in $\hat{x}$ a small circular inclusion *B*_ε_ of radius ε (see Figure 1).

Let us assume that a given shape functional (eg, the compliance) J_ε_(Ω), associated to the perturbed domain, admits the following topological asymptotic expansion:

(2) $$J_{\varepsilon }(\Omega )=J(\Omega )+f(\varepsilon )T(\hat{x})+o\left(\;f(\varepsilon )\right),$$

where $T(\hat{x})$ is the topological derivative and f(ε) is a positive function such that f(ε)→0 when ε→0^+^. Then, the topological derivative expression is obtained by passing to the limit ε→0^+^, then, we have

(3) $$T(\hat{x})=\lim_{\varepsilon \to 0^{+}}\frac{J_{\varepsilon }(\Omega )-J(\Omega )}{f(\varepsilon )}\;.$$

As shown in the work of Novotny and Sokolowski,^17^ in the case of the compliance, the topological derivative can be written as follows:

(4) $$T(\hat{x})=\sigma (\hat{x}):P:\nabla^{s}u(\hat{x})$$

with P being the fourth‐order polarization tensor and ∇^s^ u the symmetric gradient of the displacements. For isotropic materials, plane stress, and circular inclusions, (see the work of Giusti et al^20^) the polarization tensor adopts the form

$$P=-\frac{1}{2}\frac{1}{\beta \gamma +\tau_{1}}\left[\left(1+\beta )(\tau_{1}-\gamma )I+\frac{1}{2}(\alpha -\beta )\frac{\gamma (\gamma -2\tau_{3})+\tau_{1}\tau_{2}}{\alpha \gamma +\tau_{2}}(I⊗I\right)\right],$$

where I and I denote the second‐ and fourth‐order identity tensors, respectively, and

$$\alpha =\frac{1+\nu }{1-\nu },\;\beta =\frac{3-\nu }{1+\nu },\;\gamma =\frac{E^{⋆}}{E},\;\tau_{1}=\frac{1+\nu^{⋆}}{1+\nu },\;\tau_{2}=\frac{1-\nu^{⋆}}{1-\nu },\;\text{and}\;\tau_{3}=\frac{\nu^{⋆}(3\nu -4)+1}{\nu (3\nu -4)+1},$$

where values E ^⋆^ and E are the Young's modulus of the inclusion and the base material, respectively, and ν^⋆^ and ν are the corresponding Poisson's ratios. Two scenarios are considered: the first one when a hole (an inclusion with α_0_ times less young modulus and same Poisson ratio, with respect to the base material) appears in the design domain, and the second one when an inclusion of the material appears in a hole. Writing the polarization tensor as $P=P(\alpha ,\beta ,\gamma ,\tau_{1},\tau_{2},\tau_{3})$, both cases lead to the following expressions:

$$P=\left\{\begin{array}{cc} P^{+}=\; & P(\alpha ,\beta ,\alpha_{0},1,1,1)\;x\in \Omega^{+}\\ P^{-}=\; & P\left(\alpha ,\beta ,\frac{1}{\alpha_{0}},1,1,1\right)\;\;x\in \Omega^{-}, \end{array}\right.$$

where Ω^+^ and Ω^−^, fulfilling $\Omega =\Omega^{+}\cup \Omega^{-}$, stand for the domains of the base material and the inclusion, respectively. Note that α_0_>0 is a parameter small enough for modeling a void and large enough to entail invertibility properties to the stiffness matrix. In this work, we set α_0_=10^−3^. For the derivation of the polarization tensor P for the case of anisotropic materials, the readers are referred to the work of Giusti et al.^20^

### 2.2 Augmented Lagrangian Slerp algorithm

In the following, we provide a brief summary of the Slerp algorithm. We begin by stating the general optimization problem faced in topology optimization as follows:

(5) $$\begin{array}{cc} \underset{\chi }{\text{minimize}} & J(\;\chi )\\ \text{subject to:} & \int_{\Omega }\chi \;d\Omega \leq V, \end{array}$$

where $J:L^{\infty }(\Omega ,\{0,1\})\to R$ is the general cost function (normally compliance), V the volume value to achieve, and χ∈L ^∞^(Ω,{0,1}) is the characteristic function defined in this case by the level set function ψ∈C(Ω,R) as

(6) $$\chi =\left\{\begin{array}{cc} 1\; & \psi \leq 0,\\ 0\; & \psi >0. \end{array}\right.$$

Defining the following spaces:

(7) $$U:=\left\{\phi \in H^{1}(\Omega ):\phi {|}_{\Gamma_{D}}=u_{0}\right\}\;\text{and}\;V:=\left\{\phi \in H^{1}(\Omega ):\phi {|}_{\Gamma_{D}}=0\right\},$$

we can state that the characteristic function fulfills the equilibrium equation

(8) a(χ,u,v)=l(v)∀v∈V,

which corresponds to the weak form of the standard elasticity problem: find u∈U such that

(9) $$\left\{\begin{array}{c} \nabla \cdot \sigma =0\;\text{in}\;\Omega ,\\ \;\sigma =C(\chi ):\nabla^{s}u,\\ \;u=u_{0}\;\text{on}\;\Gamma_{D},\\ \sigma \cdot \;n=t\;\text{on}\;\Gamma_{N}, \end{array}\right.$$

where σ is the stress tensor and C(χ) is the elastic constitutive (fourth‐order) tensor defined as

(10) $$C(\;\chi )=\left\{\begin{array}{cc} C\; & \chi =1,\\ C^{*}\; & \chi =0. \end{array}\right.$$

Here, C and $C^{*}$ stand for the constitutive tensors of the material and the void, respectively. Thus, problem (5) may be expressed as

(11) $$\begin{array}{cc} \underset{\psi }{\text{minimize}} & J(\chi (\psi ))\\ \text{subject to:} & \;\;c(\psi )=\int_{\Omega }\chi (\psi )-V\leq 0. \end{array}$$

Following the work of Nocedal and Wright,^21^ an augmented Lagrangian scheme is applied to (11) leading to the following saddle point problem:

$$\begin{array}{lcr} \underset{\lambda }{\max\;.}\;\underset{\psi }{\min\;.}\;J\;\left(\;\chi (\psi )\right)+\lambda c(\psi )+\frac{1}{2}\rho c{\left(\psi \right)}^{2}, & & \end{array}$$

with λ being the Lagrange multiplier and ρ the penalty parameter. In the sequel, the penalty parameter is taken as ρ=1. Then, two kinds of iterations will be applied. First, a ψ updating iteration for minimizing the cost function and, then, a Uzawa‐like updating iteration^22^ for λ as

(12) $$\lambda_{n+1}=\lambda_{n}+\rho c(\psi_{n})$$

for maximizing the cost function. The update of the level‐set function ψ is based on the Slerp algorithm, proposed in the work of Amstutz and Andrä,^3^ which results in the following expression:

(13) $$\psi_{n+1}=\frac{1}{\sin\theta_{n}}\left[\sin\left((1-\kappa_{n})\theta_{n}\right)\psi_{n}+\sin(\kappa_{n}\theta_{n})\frac{g_{n}}{{\left\|g_{n}\right\|}_{L^{2}}}\right],$$

where g_n_ is the extended topological derivative, κ_n_∈[0,1] a line search‐like parameter, and θ_n_ is the angle formed by ψ_n_ and g_n_. The expression for this angle is given by

(14) $$\theta_{n}=\text{acos}\left[\frac{\left(\psi_{n},g_{n}\right)}{{\left\|\psi \right\|}_{L^{2}}{\left\|g_{n}\right\|}_{L^{2}}}\right].$$

The angle θ_n_ is usually used to define the stop criteria, when it is smaller than a certain tolerance (in this work, the results are considered converged when θ<1°).

According to the work of Amstutz,^23^ the optimality condition is fulfilled when the sign of the level‐set function coincides with the sign of the topological derivative function. To this end, the Slerp method proposes a fix‐point algorithm that seeks to iteratively obtain the following: (i) parallelism between both functions and (ii) the level‐set function with unit norm. This is precisely to prevent that the level‐set function unrestrictedly increases during iterations.

Since we are dealing with an augmented Lagrangian scheme with inequality constraints, the extended topological derivative must be computed as

(15) $$g(x)=\left\{\begin{array}{c} -T+\max\left(0,\lambda +\rho c(\psi )\right)\;\psi (x)\;\leq \;0,\\ \;T+\max\left(0,\lambda +\rho c(\psi )\right)\;\psi (x)>0, \end{array}\right.$$

with T being the topological derivative of the cost function. The augmented Lagrangian Slerp algorithm is described in Algorithm 1.

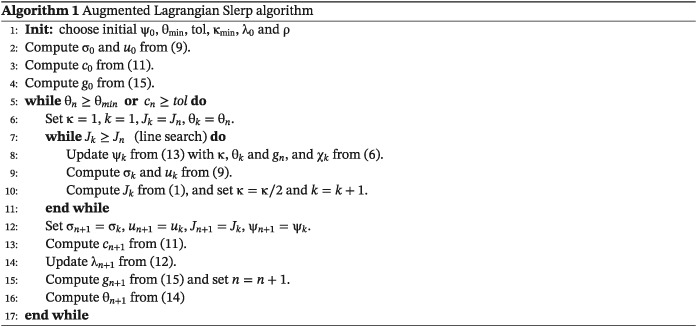

For full additional details, see other works.(3, 13, 24, 25)

### 2.3 Macroscopic and microscopic topology optimization problem

**Figure 2:**
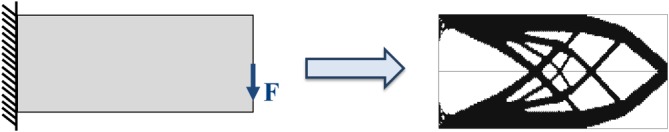
The sketch of the structural optimization problem (macroscopic topology optimization problem) for the cantilever beam case [Colour figure can be viewed at http://wileyonlinelibrary.com]

**Structural optimization problem**. Structural optimization techniques address the standard topology optimization problem with the aim of maximizing the stiffness of a body, subjected to some external forces and boundary conditions and fulfilling a maximum volume fraction constraint. Usually, the problem is written as in Equation (5), and the (mean) structural compliance is taken as the cost function. In Figure 2, a sketch of the domain in the initial configuration and the optimal topology for the classical cantilever beam illustrates the problem.

In this work, the topological derivative described in Equation (4) and the Slerp Algorithm 1 are used for solving the structural optimization problem.

**Material design problem**. Similar concept is applied in microscopic topology optimization problem. The main idea, in this case, is to endow the microstructure with particular constitutive properties and with a particular fraction volume. In this work, the objective function is the stiffness of the microstructure. Similar to problem (5), the material design problem is expressible as

(16) $$\begin{array}{cc} \underset{\chi_{\mu }}{\text{minimize}} & J(\;\chi_{\mu })\\ \text{subject to:} & \int_{\Omega }\chi_{\mu }\;d\Omega =V_{\mu }, \end{array}$$

where μ subindex is used to refer to the microstructures variables, whereas the objective function is defined by

(17) $$J(\;\chi_{\mu })=\sigma :C_{h}^{-1}(\chi_{\mu }):\sigma .$$

**Figure 3:**
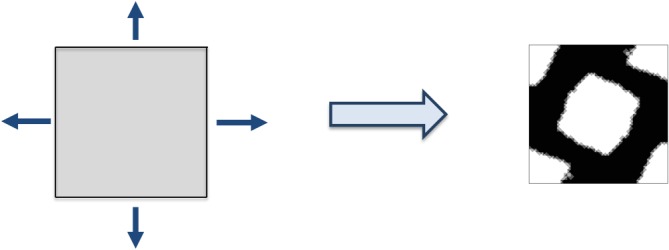
The sketch of the material design problem (microscopic topology optimization problem) for a given stress state [Colour figure can be viewed at http://wileyonlinelibrary.com]

Here, $C_{h}^{-1}$ stands for the homogenized elasticity tensor and σ is the macroscopic stress tensor—note that, in this case, this tensor is an input data and may be thought as the projection direction of the inverse of the constitutive tensor that is desired to be minimized. In Figure 3, a microcell or RVE (representative volume element) initial configuration is shown. The arrows represent the action of the (macroscopic) second‐order tensor σ into the RVE. The final topology is also shown.

For full details on how to obtain the homogenized elasticity tensor, we refer the readers to the work of de Souza Neto et al.^6^ In this work, square domains and periodic boundary conditions are considered. In the case of material design, the topological derivative for isotropic material was developed in the work of Giusti^26^ and can be expressed as Equation (4) by replacing the macroscopic stress and strain by its microscopic counterpart.

Following the proposal advocated in the work of Amstutz et al,^8^ we use also the Slerp Algorithm 1 described in Section 2.2 to solve the material design problem. As commented earlier, we shall refer to structural optimization when topology optimization is applied to the macroscale and material design when is applied to the microscale.

## 3 Multiscale Material Design Problem

**Figure 4:**
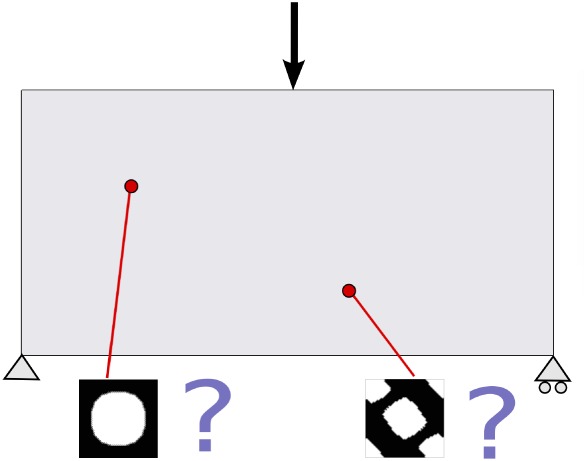
Illustration of a point‐to‐point material design problem. The topology of each microstructure is considered as the design variables of the optimization problem [Colour figure can be viewed at http://wileyonlinelibrary.com]

Suppose a fixed domain subjected to given forces and boundary conditions. The goal of the problem is to find, at every point of the macroscale, the optimal homogenized elasticity tensor $C^{h}$ through the design of its microscopic topology, ie, the topology of the unit cell associated to such a macroscopic point, such that the volume of the domain remains below a certain value and its stiffness is maximized. In Figure 4, this multiscale material design problem is illustrated.

### 3.1 Formulation of the P2P material design problem

The first approach to solve the multiscale material design problem is the P2P material design approach, in which the topologies of the microstructures can freely vary over the domain, ie, neighbouring points at the macroscale may feature significantly distinct microstructures. As we shall discuss later, this fact renders this approach nonviable because of two reasons. Firstly, from a mathematical point of view, the use of periodic boundary conditions for the unit cell tacitly presumes that the macroscopic body is constructed by spatial repetition of similar unit cells; and secondly, from a practical point of view, a structure made of a material whose microtopology virtually changes from point is hardly manufacturable. Nevertheless, we present the formulation of the P2P problem because it will serve as benchmark for assessing the quality of the results obtained when manufacturability constraints are introduced—this will be discussed in the CB approach of Section 3.3.

In mathematical terms, the P2P material design problem can be formulated as

(18) $$\begin{array}{cc} \underset{\sigma ,\chi_{\mu }}{\text{minimize}} & \;\int_{\Omega }\sigma :C_{h}^{-1}(\chi_{\mu }):\sigma \;d\Omega \\ \text{subject to:} & \;\int_{\Omega_{\mu }}\chi_{\mu }d\Omega_{\mu }\leq V_{\mu },\\ & \;\;\;\nabla \cdot \sigma =0,\\ & \;\text{+ Boundary conditions.} \end{array}$$

Note that the macroscopic stress tensor σ depends on χ_μ_ through the homogenized elasticity tensor. Notice also that the cost function and the stress tensor are defined at the macroscale, whereas the design variables (characteristic function χ_μ_) are defined at the microscale. In terms of the design variables (σ and χ_μ_), the problem is highly nonlinear in both the cost function and the equilibrium equation.

Although it is not standard, the problem has been formulated by adding explicitly the equilibrium equation as a constraint and the stress tensor as a design variable. Following the work of Allaire,^5^ it helps on understanding the essence of the further explained alternate direction algorithm. Regarding the computational cost of the optimization problem, it becomes prohibitive after the finite element discretization at both scales, since the total number of design variables is equal to the product of the numbers of macroscopic and microscopic Gauss points.

### 3.2 Brief review of the P2P approach

To alleviate the computational burden associated to the P2P material design problem described in the foregoing, we use here the strategy proposed in our recent work.^13^ For completeness, we succinctly review in the following the main ingredients of this approach.

The first ingredient is to adopt an alternate direction strategy for solving problem (18) in the sense that, first, for a given distribution of microscopic topologies, an equilibrium iteration is solved, obtaining the stresses σ, and second, with these stresses, a new characteristic function χ_μ_(a new distribution of microscopic topologies) is obtained.

The second ingredient relies on separability properties for this last iteration. Since each characteristic function is local, it means that it is uncoupled from the other RVE characteristic function; accordingly, instead of a large minimization iteration for all characteristic function, we solve a sequence of local optimization problems (one for each RVE). In other words, one large optimization problem is divided into many small optimization problems.

The third idea is related to computational efficiency aspects. Basically, instead of solving a multiscale material design problem (see Section 3.2.2) for all RVEs in each iteration, a precomputation of all material design problems is considered. Specifically, for each possible macroscopic stress tensor σ (which represents the input data), a material design problem is solved, ie, a microstructure topology is designed. As a result, a microstructure database, what we call *Computational Vademecum*, is created in an offline process. In doing so, the computational effort of simultaneously solving the multiscale and the material design problem is split into a perhaps costly offline step, which is performed only once, and a relatively inexpensive “online” optimization. In this online optimization, the homogenized optimal constitutive tensor associated to a given input macrostress is retrieved from the database—hence the computational savings.

#### 3.2.1 Computational Vademecum

**Figure 5:**
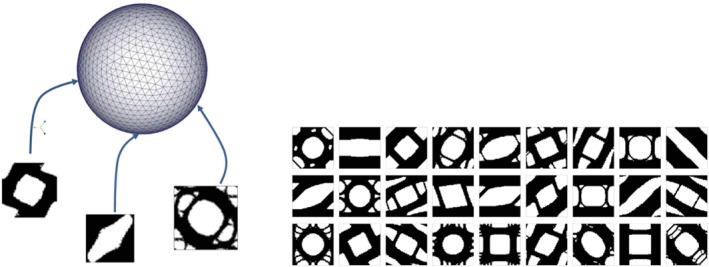
Computational Vademecum [Colour figure can be viewed at http://wileyonlinelibrary.com]

The idea behind the construction of what we have called the “Vademecum” of optimal structures is to try to precompute the optimal microtopology for a sufficiently rich representative sample of input macroscopic stresses. Therefore, the first question one has to face is how to efficiently sample, ie, discretize, the stress space. This question has been already addressed in our recent work.^13^ It is shown therein that, in the small strain regime, the modulus of the input stress has no influence on the final topology. This means that, in a two‐dimensional problem, the space of all possible configurations lies on a sphere. Indeed, expressing the second‐order tensor σ in Voigt notation, we have that

$$\sigma =\left[\begin{array}{c} \sigma_{x}\\ \sigma_{y}\\ \sigma_{xy} \end{array}\right]=\left[\begin{array}{c} \cos(\phi )\cos(\theta )\\ \sin(\phi )\cos(\theta )\\ \sin(\theta ) \end{array}\right],$$

with ϕ and θ being the spherical coordinates. Thus, the parametric domain can be represented by the unit sphere (see Figure 5).

Furthermore, in the small strain regime, ie, the scope of this work, the sign of the stresses does not play any role on the final topology either, in the sense that the optimal microstructure for a tensile stress state, eg, [*a*,0,0]^*T*^ would be the same as that of a compression stress state 1Needless to say, this conclusion should be revised when extending the Vademecum idea to large strains.[−*a*,0,0]^*T*^. These and other considerations allow one to significantly reduce the computational burden associated to the construction of the Vademecum, since only one eighth of the whole sphere has to be considered for the computations (see more details in our recent work^13^).

**Figure 6:**
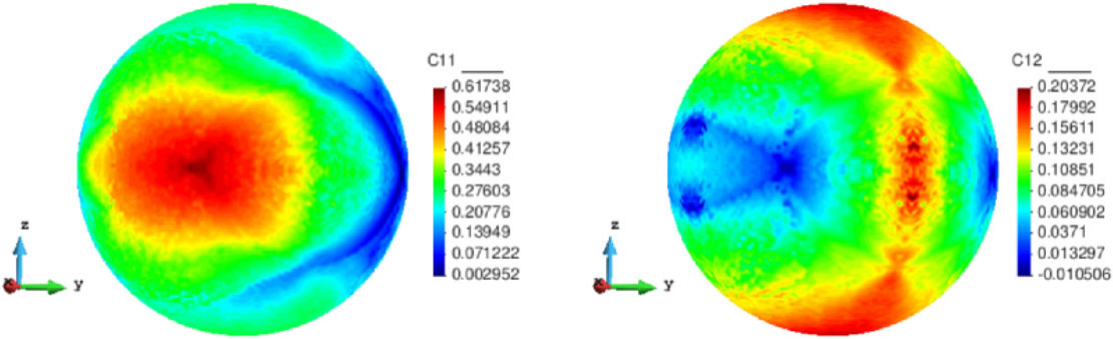
Maps of the components $C_{h11}$ and $C_{h12}$ of the elasticity matrix on the unit‐radius spherical parametric domain

Some examples of these optimized RVE typologies with volume fraction of *V*_μ_=0.6 with a triangular structured mesh of 6400 elements are displayed in Figure 5. Apart from the topologies, the homogenized constitutive tensor of all sampled stresses is to be saved in a database, since this tensor is the output of the material design problem (16) and it is necessary for solving the equilibrium problem (9). By way of illustration, we show in Figure 6 the contour plot of entries $C_{h11}$ and $C_{h12}$ of such a tensor on the unit sphere.

Note that, in general, problem (16) can be parameterized by many different parameters, eg, the Young' modulus and Poisson ratio of both the weak and stiff materials, the fraction volume, or the macroscopic stress. In the presented method, the Computational Vademecum is only parameterized by the macroscopic stress. Further research should be done to increase the dimension of the parametric domain

#### 3.2.2 Strategy and algorithm

The strategy for solving the P2P material design problem relies on the alternate direction algorithm, widely used in literature (see the work of Allaire^5^). In Algorithm 2, we present full details of such an algorithm.

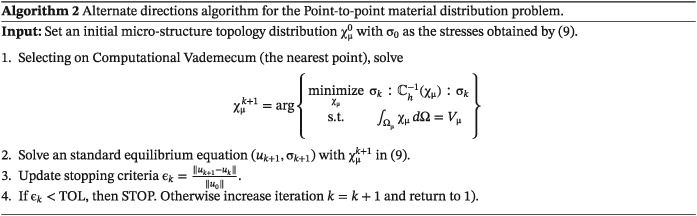

The discretization of the Computational Vademecum leads to a discontinuous compliance function and a discontinuous homogenized constitutive tensor. To deal with this, the strategy (step 1) is to select the nearest point (microstructure) in the regular spherical mesh of the Computational Vademecum. This entails almost negligible computational cost in comparison with solving an equilibrium equation.

#### 3.2.3 Numerical example for the P2P material design problem

**Figure 7:**
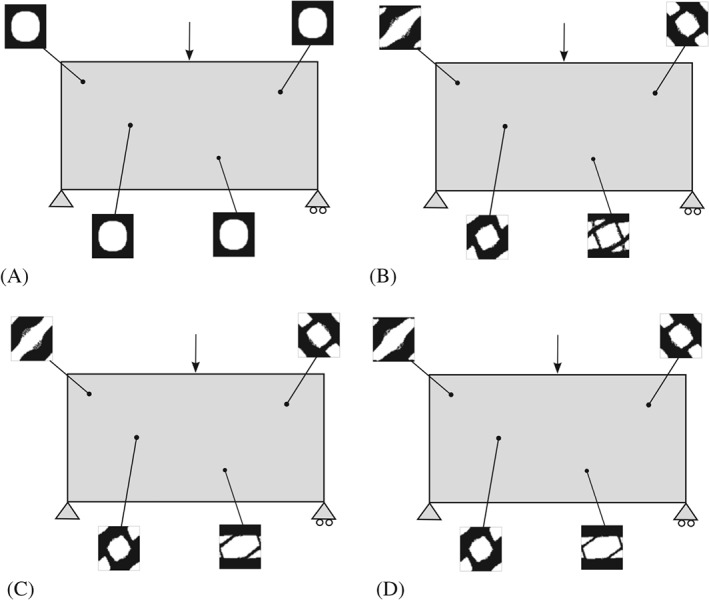
Point‐to‐point material design problem for the a bending beam. The homogeneous material distribution used in the first iteration and the corresponding iterations are shown. The material distribution is allowed to be designed point by point. A, Iteration 1; B, Iteration 2; C, Iteration 3; D, Final iteration

To illustrate the performance of the P2P approach, we show in Figure 7 an example consisting of a rectangular domain hinged at its bottom corners and subjected to a concentrated unit load at its top middle. The domain is discretized with a nonstructured mesh of 5056 linear triangular element, the volume fraction is V_μ_=0.6, and the tolerance for stopping the iterative procedure is set to T O L=10^−3^. On the other hand, the microscopic domain is discretized with a mesh of 6400 linear triangular elements.

In Figure 7, we show the initial condition with a microstructure (square with a hole) satisfying the desired volume. In addition, intermediate iterations and the optimal material distribution with some representative (not symmetrically selected) microstructures are presented. Note that the obtained result offers a symmetric solution and that nonintuitive and significantly different microstructures topologies are obtained in the solution of the P2P material design problem.

### 3.3 CB approach (design by components)

One may argue that alternate direction algorithm 2 theoretically solves the P2P material design problem (18). However, the results of the P2P material design approach present relevant manufacturing limitations. Designing the material of a structure featuring different microstructure P2P seems unrealistic. Since this work aims at presenting clear realistic results, some additional constraints in problem (18) have to be introduced. This gives rise to the hereafter termed CB material design problem.

**Figure 8:**
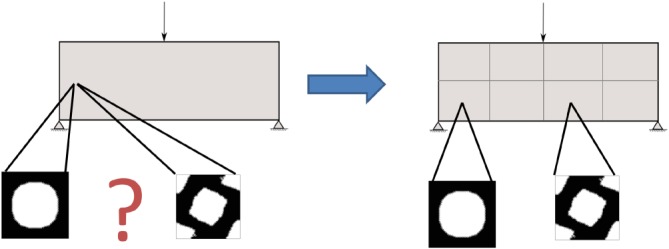
Representation of how the point‐to‐point material design problem becomes the CB material design problem. The domain is now divided in subdomains (components), and the microstructure is imposed to be homogeneous in each subdomain to fulfill manufacturing constraints [Colour figure can be viewed at http://wileyonlinelibrary.com]

In order to establish such manufacturing constraints, the macrostructure is divided in subdomains (or components) and the same microstructure is imposed in each subdomain. In optimization terms, this can be formulated as

(19) $$\begin{array}{cc} \underset{\sigma ,\chi_{\mu_{i}}}{\text{minimize}} & \;\sum_{i}^{n}\int_{\Omega_{i}}\sigma :C_{h}^{-1}(\chi_{\mu_{i}}):\sigma \;d\Omega \\ \text{subject to:} & \;\int_{\Omega_{\mu_{i}}}\chi_{\mu_{i}}\leq V_{\mu }\;d\Omega ,\\ & \;\nabla \cdot \sigma =0,\\ & \;\text{+ Boundary conditions,} \end{array}$$

where Ω_i_ stands for the volume of each subdomain ( $\cup_{i}^{n}\Omega_{i}=\Omega$). Figure 8 depicts roughly how the P2P material design problem becomes the CB material design problem.

#### 3.3.1 Suboptimal reformulation

From the optimization point of view, the manufacturability constraints impose the same microstructure in each component. This turns into a decrease of the number of design variables. Thus, the *CB material design problem* (19) can be seen a priori more affordable than the *P2P material design* problem. However, the opposite happens. On the one hand, the decoupling, explained in Section 3.2, no longer holds. On the other hand, the number of design variables increase simultaneously with the number of components.

These reasons give rise to propose, as a remedy, a reformulation of the *CB material design problem* (19). In mathematical terms, we propose the following (suboptimal but feasible) problem:

(20) $$\begin{array}{cc} \underset{\sigma ,\chi_{\mu_{i}}\in V}{\text{minimize}} & \;\sum_{i}^{n}\int_{\Omega_{i}}\sigma :C_{h}^{-1}(\;\chi_{\mu_{i}}):\sigma \;d\Omega \\ \text{subject to:} & \;\nabla \cdot \sigma =0,\\ & \;\text{+ Boundary conditions,} \end{array}$$

where V corresponds to the *Vademecum* space as follows:

(21) $$V=\left\{\chi_{\mu }\in L^{\infty }(\Omega ,\{0,1\})\;\;|\;\;\chi_{\mu }\;\text{solves problem (16)}\right\}$$

and *n* stands for the number of subdomains. In other words, the possible microstructure topologies are going to be searched in the *Computational Vademecum*, ie, in the database generated offline, even if they are not optimal with respect the current stress state of the subdomain.

Instead of looking for the optimal microstructure topologies of a subdomain in the all possible microstructure topology set, we rather prefer seeking it in the already computed Vademecum set. Certainly, as a disadvantage, it leads to suboptimal solutions. Nevertheless, as an advantage, the computations of solving all the microscopic topology optimization problems (in each component in each iteration) are avoided.

Defining $\chi_{\mu }^{p2p}$ as the solution of the *P2P material design problem* (18), $\chi_{\mu }^{man}$ as the solution of *CB material design problem* (19), and $\chi_{\mu }^{com}$ as the solution of the *suboptimal* *CB material design problem* (20), the cost function satisfies the following inequalities:

(22) $$J\left(\chi_{\mu }^{\;p2p}\right)\leq J(\chi_{\mu }^{man})\leq J\left(\chi_{\mu }^{\;com}\right).$$

Since the *CB material design problem* (19) will not be tackled, hereafter the *suboptimal* *CB material design problem* will be termed *CB material design problem* indistinguishably.

#### 3.3.2 Strategy and algorithm

As proposed in the *P2P material design problem*, an alternate direction algorithm is used for solving the *CB material design problem* (20). The resulting strategy mainly mimics, roughly speaking, the one proposed in the *P2P material design problem*. It takes advantage of the separability property (the subdomains play the role of the Gauss points) and the computational savings due to the microstructure database (*Computational Vademecum*).

However, for the material design iteration of Algorithm 2, an slightly different problem must be solved. More specifically, for a stress state value, instead of problem (16), the microstructure topologies must solve the following problem:

(23) $$\underset{\chi_{\mu_{i}}\in V}{\text{minimize}}\int_{\Omega_{i}}\sigma :C_{h}^{-1}(\;\chi_{\mu_{i}}):\sigma \;d\Omega .$$

In turn, since the *Computational Vademecum* space V (unit sphere) can be parameterized in two variables (θ and ϕ), problem (23) can be rewritten in the following terms,

(24) $$\underset{\theta ,\phi }{\text{minimize}}\int_{\Omega_{i}}\sigma :C_{h}^{-1}(\theta ,\phi ):\sigma .$$

Note that since we are using an alternate direction algorithm, when solving problem (24), the stress variables are frozen (fixed direction). Consequently, they are independent of θ and ϕ and can be considered given data of problem (24). Thus, an optimization problem of two variables must be solved for each subdomain. More specifically, the optimization problem consists in finding in the unit sphere (*Computational Vademecum*), the point (microstructure) that minimizes the compliance.

In our case, we try all the possible constitutive tensors of our database, selecting the one that provides the minimum value of the cost. Certainly, better and more efficient procedures can be proposed. However, since the value of the constitutive tensor is already stored, only simple matrix‐vector product must be computed. Our experience shows that this computational operation is much less significant than solving the FEM system of equations which, in computational terms, represents the bottleneck of the problem. This strategy is summarized in Algorithm 3.

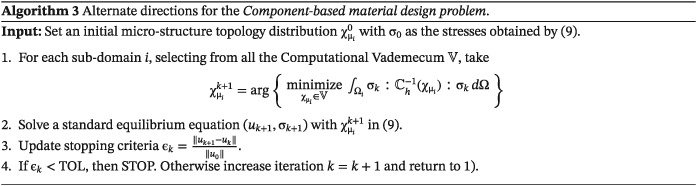

### 3.4 Numerical results of the CB material design problem

Two numerical examples are performed to illustrate the performance of the *CB material design* strategy.

**Figure 9:**
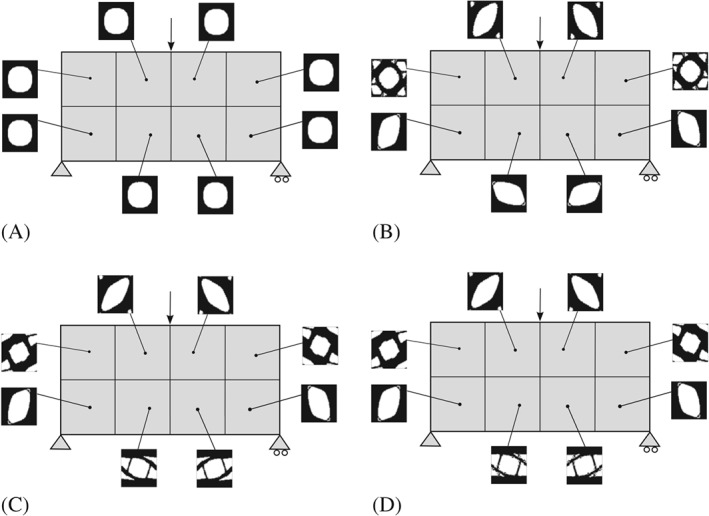
Component‐based material design problem applied to the bending beam example. Homogeneous material distribution is used in the first iteration and different microstructures appear during the iteration process. The material is not designed P2P; rather, the same microstructure is employed in a given subdomain (fulfilling manufacturing constrains). A, Iteration 1; B, Iteration 2; C, Iteration 3; D, Final Iteration

**Bending beam.** In this example, we present the bending beam for the *CB* approach. In both cases, the domain is discretized by a nonstructured mesh of 5056 linear triangular element. As seen in Figure 9, the domain is regularly partitioned in 8 subdomains. No symmetric constraints are added in the problem and the initial material distribution is taken as in the *P2P* case. Additionally, intermediate and final iterations are also shown. Note that in this case, the same microstructure is used in the whole subdomain bringing a *CB* design.

**Figure 10:**
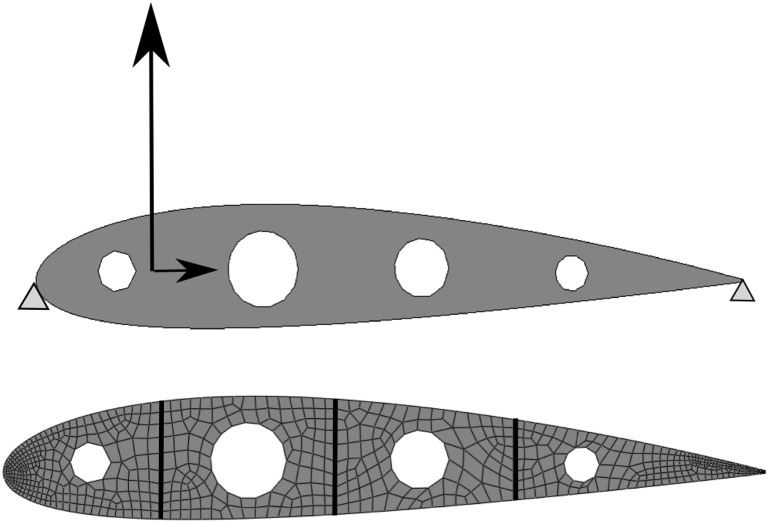
Boundary conditions and discretization of the aerodynamic profile with an unstructured mesh. The partition of the domain by components is also shown.

**Aerodynamic profile.** We consider an aerodynamic profile—standard wing rib—as a second numerical example. Although the bending forces and the instabilities such as buckling strongly determine the optimal design, we assume for the sake of simplicity plane stress state and small strains. In addition, the aerodynamic forces (Lift *L*=10 and Drag *D*=1) are modeled as singular forces applied on the aerodynamic center (see Figure 10). The aerodynamic profile is partitioned in 4 subdomains (see Figure 10).

**Figure 11:**
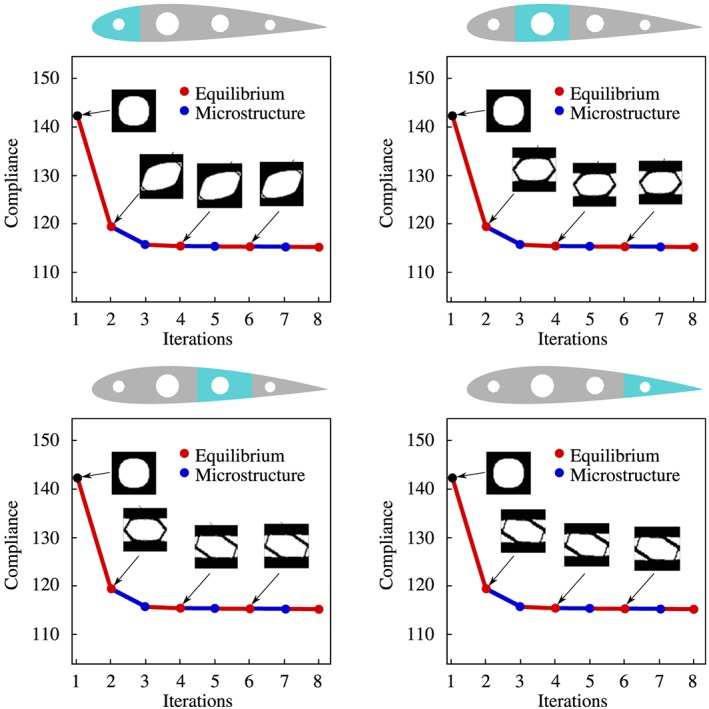
Aerodynamic profile example. Representation of the microstructure history iterations of the 4 components of the structure. The cost is minimized with respect to σ in blue and with respect to χ_μ_ (microstructure) in red. This representation of the iterations illustrate the spirit of the alternate direction algorithm

As a starting point, microstructure topologies with a circular hole fulfilling the desired fraction value V_μ_=0.6 are considered. Thus, the initial iteration corresponds indeed to a feasible iteration. In order to shed light on the alternate direction algorithm 3 of the CB material design problem, we show, in Figure 11, the descent different directions in different columns. In the first column, we solve an equilibrium equation which, in optimization terms, leads to minimize the compliance with respect to the stresses σ. The decrease in the cost function due to the equilibrium is represented in blue on the cost function line. On the contrary, the decrease in the cost when selecting the optimal microstructures (minimizing with respect to χ_μ_) is shown in red and it is represented on the second column of Figure 10.

Note that the algorithm converges extremely fast, almost one iteration of minimizing with respect to χ_μ_ is needed to decrease the major part of the cost function. In this case, the optimal *CB material design* solution provides an increasing of around a 16% of the stiffness with respect to the initial iteration by providing appropriate microstructures on each component.

**Figure 12:**
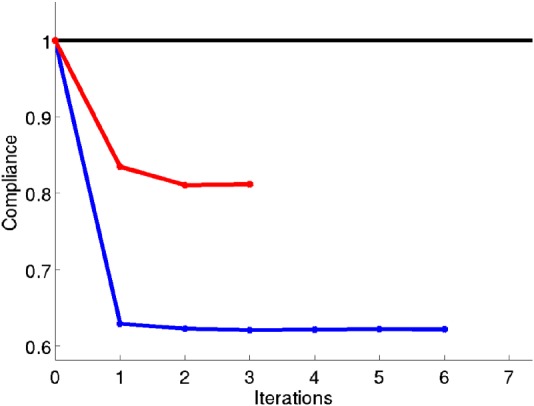
Compliance comparison between point‐to‐point (blue) and component‐based material design approach (red) for the bending beam example

**Comparison between P2P and CB material design problem.** At this point, it is convenient to compare the *P2P* and *CB material design* approaches. Both approaches tackle the same optimization problem with the difference that the former includes more number of design variables. According to Equation (22), this fact should result into smaller values of the cost function. In Figure 12, the cost function for both approaches, in the case of the bending beam example, is depicted. As expected, the *P2P material design* approach achieves smaller values of the cost function.

In the case of *P2P material design approach,* the compliance decreases around 35%, whereas in the case of *CB material design approach*, the compliance decreases around 18%. In both cases the stopping criteria is taken as *ε*_*k*_=10^−2^. Note that, although a strong nonlinearity is faced, convergence is achieved in just few iterations. Additionally, it is worth stressing that, due to the Computational Vademecum, these two numerical examples have been solved in less than 5 minutes of computation with a standard PC (3.40 GHz processor in a 64‐bit architecture) in a MATLAB© environment.

### 3.5 Consistency and efficiency

As mentioned in problem (20), the main idea of the *CB material design approach* lies on assuming homogeneous material distribution in each subdomain. In principle, the subdomain partition is a priori decided by the user depending on the particularities of the problem. However, it seems clear that, for a fixed domain, the number of variables of the optimization problem increases insofar as the number of subdomains increases, and consequently, the objective function may decrease.

Therefore, the following question naturally arises at this juncture of the discussion: *does a sequence of problems solved by the CB material design approach, in which the number of subdivisions increases, converge to the solution of the problem solved by the P2P material design approach when the size of the subdomains coincides with the size of the elements?* In other words, if the *CB material design approach* is applied to a problem with the size of the elements of the size of the subdomain and its compliance is compared with the compliance obtained by the *P2P* *material design* approach, do we obtain the same values? If this is the case, we say that the *CB material design* approach is a consistent approach.

Similarly, a second question emerges in this regard: *if, in the case of the study, the size of the subdomain can be arbitrary decided, which size should be selected and how will it influence on the cost function?* This second question, later explained, is related with the efficiency concept.

**Consistency.** Let us study a sequence of *CB material design* problems in which the number of subdomains increases so that, eventually, subdomains coincide with the elements of the underlying finite element mesh. Then, we compare with the solution of the same problem solved by the *P2P material design* approach. To this end, we apply both methodologies to the bending beam example. The domain is discretized with a regular coarse mesh of 128 $Q_{1}$ elements.

**Figure 13:**
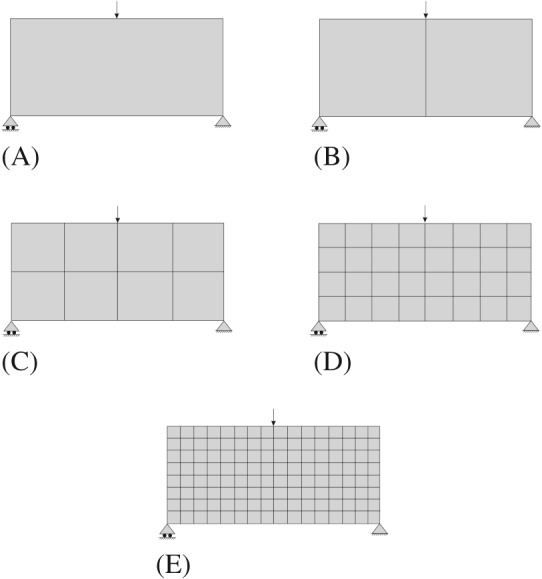
A sequence of problems, with different number of subdomains, is represented. The aim is to study the convergence of the component‐based material design approach to the P2P material design approach. A, One Subdomain; B, 2 Subdomains; C, 8 Subdomains; D, 32 Subdomains; E, 128 Subdomains

**Figure 14:**
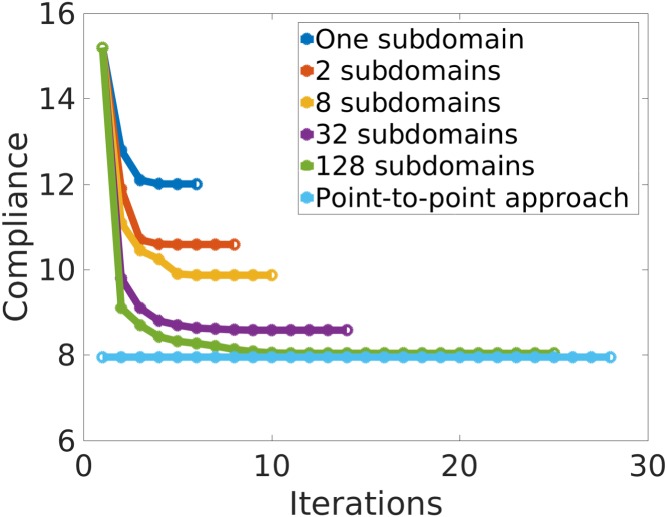
Representation of the compliance values as a function of the number of iterations for the sequence of subproblems shown in Figure 13. In sky‐blue, the compliance for the P2P material design approach is depicted. As expected, the P2P material design solution behaves as a lower bound of the component‐based material design approach

The sequence of problems with different subdomains starts by considering the problem with only one subdomain (homogeneous material distribution over all the domain). Then, it is divided in 2, 8, 32, and 128 subdomains. A full illustration of the sequence of problems used for the computations is shown in Figure 13. Regarding the boundary conditions, a concentrated force is applied in the middle top part of the domain and it is supported on the bottom corners. The ratio length/height of the rectangular domain is 2. After solving the sequence of problems, we depict in Figure 14 the values of the compliance.

We observe that in each case, the minimum values of the compliance decrease monotonically as the number of subdomains increases. It should be noted that the relatively large number of iterations required to reach the optimal solution should not be worrisome since, in real applications, the number of subdomains is not expected to be high. Clearly, this behavior is a consequence of the increment of design variables produced by the increment of subdomains.

**Figure 15:**
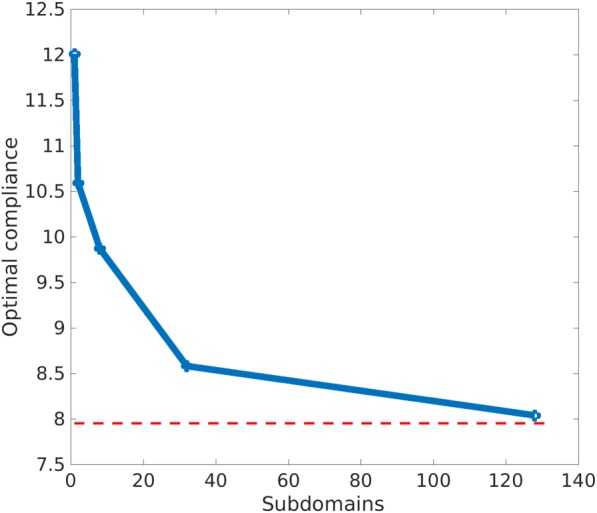
Convergence of the component‐based material design approach to the P2P material design approach. The compliance values are represented in blue for the sequence of problems using the component‐based material design approach shown in Figure 13. In red, the optimal value of the compliance obtained by the P2P material design approach. Note the convergence of both approaches as the number of subdomains converges to the number of elements [Colour figure can be viewed at http://wileyonlinelibrary.com]

The compliance of the *P2P material design approach* is also included in Figure 14 as a reference. This solution can be interpreted as a lower bound for the *CB material design approach*. This result illustrates the inequality constraint stated in expression (22). Note that the compliance value obtained in this case of 128 subdomains, in which the elements and subdomains coincide, is not equal to the value of the *P2P material design approach*. The reason lies in the fact that, in the 128 subdomains, the four Gauss points of each $Q_{1}$ element takes the same microstructure while this is not the case in the *P2P material design approach*. Hence, it achieves smaller values in the cost. In Figure 15, we can observe how the solution of the *P2P material design* approaches converges to the *CB material design* approach.

**Efficiency.** We define the efficiency parameter *η*_eff_ as the ratio between the absolute value of the optimal solution obtained by the *P2P material design* $\chi_{\mu }^{\;p2p}$ and the absolute value of the suboptimal solution obtained by the the *CB material design* $\chi_{\mu }^{com}$ with a fixed number of subdomains, ie,

(25) $$\eta_{eff}=\frac{\left|\;J\left(\chi_{\mu }^{com}\right)\right|}{\left|J\left(\chi_{\mu }^{p2p}\right)\right|}x100.$$

**Figure 16:**
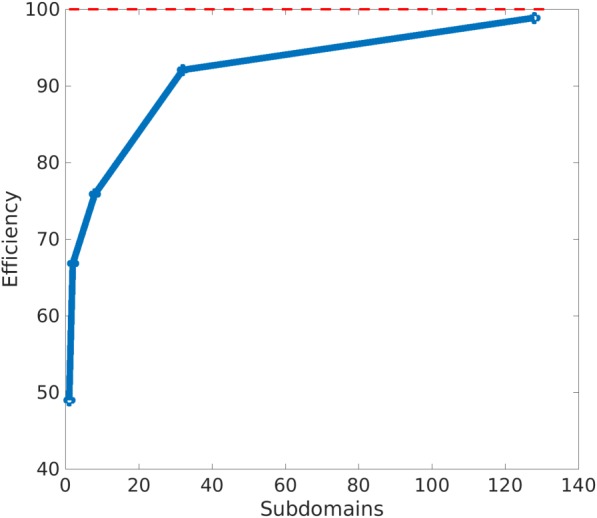
Representation of the efficiency parameter η_eff_ as a function of the number of subdomains. With just a small number of subdomains, the solution is close to the optimal (theoretical) solution [Colour figure can be viewed at http://wileyonlinelibrary.com]

According to the compliance inequalities stated in relation (22), the efficiency parameter takes always positive values. In Figure 16, we depict the variation of the efficiency parameter with the number of subdomains.

We first observe how the efficiency value increases when the number of subdomains increases—although at the expense of adding manufacturing limitations. Thus, the problem is governed by a tradeoff between efficiency and manufacturing aspects. Figure 16 shows the dependency between the efficiency and the number of subdomains. Notice that, in Figure 16, with only one subdomain, ie, by just deciding the optimal microstructure used in the whole domain, we obtain almost a 50% of efficiency. Note that the efficiency considerably increases with a slight increase of the number of subdomains. This result shows that with just a small number of subdomains, the solution is close to the optimal (theoretical) value.

## 4 Multiscale Topology Optimization

We turn our attention now to what we call the “multiscale topology optimization” problem. In this problem, one seeks to maximize the stiffness of the structure by designing, at the same time, the structural topology itself and the microtopology (for each macroscopic Gauss point). Similar to the problem encountered in the preceding sections, the multiscale topology optimization may result into a *P2P* or *CB* topological optimization.

### 4.1 P2P multiscale topology optimization

The main idea in the P2P multiscale approach relies on solving a macrostructural optimization problem and, simultaneously, addressing a *P2P material design* problem in each iteration. This procedure is possible because of the gains in computational efficiency achieved by means of the Computational Vademecum strategy. In mathematical terms, it can be understood as a generalization of the alternate direction algorithm, in which a new variable χ is added. This variable corresponds to the standard macrostructure topology optimization design variable. More specifically, due to the alternate direction algorithm, during the iterations, we can uncouple the macroscopic topology problem and the *P2P material design* problem. Note that, in this multiscale topology optimization problem, only suboptimal solutions will be found due to the nonconvexity of the problem. For solving the *P2P material design* problem, we exploit the algorithm already discussed in Section 2.

**Formulation.** Formally, the two‐scale topology optimization problem can be presented as follows:

(26) $$\begin{array}{cc} \underset{\sigma ,\chi_{\mu },\chi }{\text{minimize}} & \;\int_{\Omega }\chi \sigma :C_{h}^{-1}(\chi_{\mu }):\sigma \;d\Omega \\ \text{subject to:} & \;\int_{\Omega_{\mu }}\chi_{\mu }d\Omega_{\mu }\leq V_{\mu }\\ & \;\int_{\Omega }\chi d\Omega \leq V\\ & \;\nabla \cdot \sigma =0,\\ & +\text{Boundary conditions.} \end{array}$$

The incorporation of the characteristic function χ extends the *P2P material design* problem to the *P2P multiscale topology optimization* problem. The comments made when dealing with the material design problem still hold in its multiscale counterpart. Needless to say, the *multiscale topology optimization* problem presents stronger nonlinearities and involves computations that are more time consuming than that in the material design case. The number of design variables are of the order of the macroscopic Gauss points times the microscopic Gauss points for χ_μ_ plus the order of macroscopic Gauss points of σ and χ. Thus, in this problem, finding remedies to tackle the inherently unaffordable computational cost of *multiscale topology optimization* problem is mandatory.

**Strategy and algorithm.** To this end, we propose a particular alternate direction algorithm to solve problem (26). More specifically, for each macroscopic topology optimization iteration, we solve a *P2P material design* problem. Thus, the proposed algorithm alternates nonuniformly between directions. In mathematical terms, we can reformulate problem (26) as

(27) $$\begin{array}{cc} \underset{\chi }{\text{min.}} & \;\underset{\sigma ,\chi_{\mu }}{\text{min.}}\;\int_{\Omega }\chi \sigma :C_{h}^{-1}(\chi_{\mu }):\sigma \;d\Omega \\ & \text{s. t.}\;\int_{\Omega_{\mu }}\chi_{\mu }d\Omega_{\mu }\leq V_{\mu }\\ & \;\int_{\Omega }\chi d\Omega \leq V\\ & \;\nabla \cdot \sigma =0,\\ & \;+\text{Boundary conditions.} \end{array}$$

Thus, in solving the preceding optimization problem, we seek to find, for a fixed macroscopic topology χ, the solution of the inner $\underset{\sigma ,\chi_{\mu }}{\text{min}}$ problem (or loop) and then compute an iteration of the $\underset{\chi }{\text{min}}$ problem (or outer loop) in the Slerp algorithm. Therefore, the algorithm can be devised as an appropriate combination of Algorithm 1 and the *P2P material design* Algorithm 2. Full details are described in Algorithm 4.

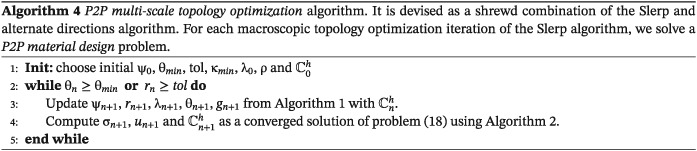

It is worth stressing that this strategy is only possible because of the reduced time‐consuming computation (due to the *Computational Vademecum*) needed for solving the *P2P material design* problem.

Note that, in the proposed algorithm, the microstructure fraction volume is enforced to be homogeneous. In the case of not enforcing the microstructure fraction volume, the design variable space may increase, and consequently, new local minimums could appear. This is left for future research.

### 4.2 CB multiscale topology optimization

Next step is to consider manufacturing constraints. Emphasis will be placed on how to diminish the inherent complexity of such a problem. To tackle it, we mimic the remedies proposed in the *CB material design* problem described in (20) for considering manufacturing constraints. Thus, on the one hand, the *material design* problem is constrained to adopt, in each subdomain, the same microstructure. On the other hand, since the microstructures are limited to the ones computed in the database, we solve a modified and suboptimal problem.

**Formulation.** More concretely, the *CB multiscale topology optimization* can be formally formulated as follows:

(28) $$\begin{array}{cc} \underset{\sigma ,\chi ,\;\;\chi_{\mu_{i}}\in V}{\text{minimize}} & \;\sum_{i}^{n}\int_{\Omega_{i}}\chi \sigma :C_{h}^{-1}(\;\chi_{\mu_{i}}):\sigma \;d\Omega \\ \text{subject to:} & \;\int_{\Omega }\chi d\Omega \leq V,\\ & \;\nabla \cdot \sigma =0,\\ & \;+\text{Boundary conditions,} \end{array}$$

where *n* stands again for the number of subdomains. Although the microstructure topology is constant in each subdomain, the optimization problem still considers a huge number of design variables. Note that, in the *CB material design problem*, the microstructure is requested to pertain to the Computational Vademecum.

**Strategy and algorithm.** A natural way of extending the *CB material design* algorithm 3 to the *CB multiscale topology optimization* problem is to adapt the *P2P multiscale topology optimization*(Algorithm 4). In fact, the major difference lies on the way that the microscopic topologies $\chi_{\mu_{i}}$ are determined. Indeed, these variables, rather than solved from the *P2P material design* problem (18) with Algorithm 2, they will be determined by solving the *CB material design* problem (20) with Algorithm 3. Note that again, this algorithm, alternates nonuniformly between directions. In mathematical terms, it consists of rephrasing problem (28) as

(29) $$\begin{array}{cc} \underset{\chi }{\min.} & \underset{\sigma ,\chi_{\mu_{i}}\in V}{\min.}\;\sum_{n}^{i}\int_{\Omega_{i}}\chi \sigma :C_{h}^{-1}(\chi_{\mu_{i}}):\sigma \;d\Omega \\ & \;\text{s. t.}\;\int_{\Omega }\chi d\Omega \leq V,\\ & \;\nabla \cdot \sigma =0,\\ & \;+\text{Boundary conditions.} \end{array}$$

Thus, similar to the P2P version, the problem is, first, to solve and converge the inner $\underset{\sigma ,\chi_{\mu_{i}}\in V}{\text{min.}}$ problem (or loop) and then compute an iteration of the $\underset{\chi }{\text{min.}}$ problem (or outer loop) in the Slerp algorithm. This final strategy is presented in Algorithm 5.

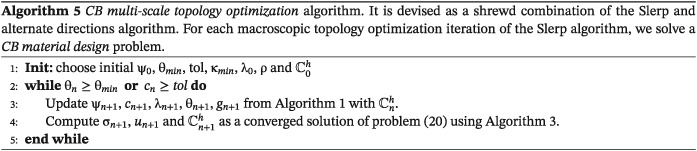

Again, the Computational Vademecum strategy allows this problem in affordable times.

### 4.3 Numerical results

In the following, in order to asses the performance of the proposed strategies, we perform a comparison between the optimal topologies obtained with the *P2P*, *CB* *multiscale topology optimization* and the standard macroscopic topology optimization problem.

**Figure 17:**
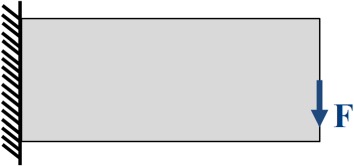
Schematic drawing of a cantilever beam [Colour figure can be viewed at http://wileyonlinelibrary.com]

**Cantilever beam.** We begin with the cantilever beam example. The domain is discretized with a structured mesh of 10 704 linear triangular elements. As shown in Figure 17, a concentrated force in the right side is applied, whereas homogeneous Dirichlet boundary conditions (clamped) on the left side are imposed. The simulation starts with full material everywhere, the fraction volume is enforced to be *V*=0.6, and we take λ_0_=0 and ρ=1. The stop criterion is taken as ε_θ_=1° and *T* *O* *L*=10^−3^.

**Figure 18:**
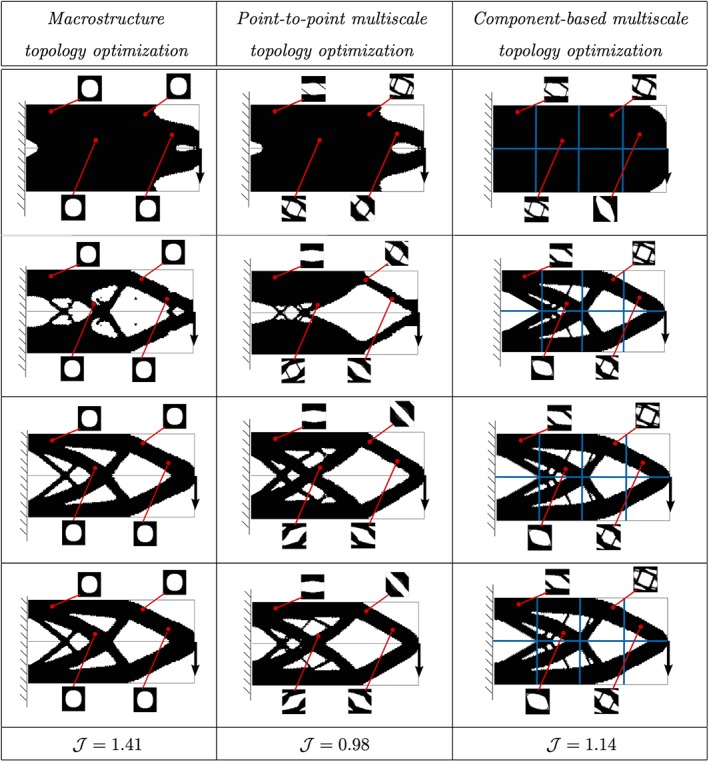
Multiscale topology optimization of the cantilever beam. In the left column, the macroscale topology optimization is solved. In the middle column, the P2P multiscale topology optimization is solved. In the right column, the CB multiscale topology optimization is solved. Cost function values are shown in the last row [Colour figure can be viewed at http://wileyonlinelibrary.com]

In Figure 18, we evaluate the improvement of the compliance of each approach. First, in the left column, fixed microstructure over all the domain (homogeneous) is considered, ie, a standard *macrostructure topology optimization* problem is solved. Secondly, in the middle column, apart from the macro, the material design problem is considered point to point, ie, the *P2P multiscale topology optimization* problem is solved. Finally, in the third column, we show the solution of the *CB multiscale topology optimization* problem. To establish a fair comparison, in the *macrostructure topology optimization* case, we have considered a microstructure with a feasible fraction volume *V*_μ_=0.6.

Regarding the different algorithms, the left column shows how Algorithm 1 solves problem (11), the middle one shows how Algorithm 4 solves problem (26), and the right one shows how Algorithm 5 solves problem (28). In rows, we show some intermediate iterations and the final one.

Owing to the computational savings already mentioned in the section (no microstructure computations are considered in the online process), we could solve all the problems in less than 10 minutes of computation with a standard PC (3.40 GHz processor in a 64‐bit architecture) in a MATLAB© environment. Interestingly, observe that the microstructure topology design tries to mimic the macrostructure topology. Likewise, observe that the microscopic topology tends to adopt patterns that resemble those of the macrostructure topology.

**Figure 19:**
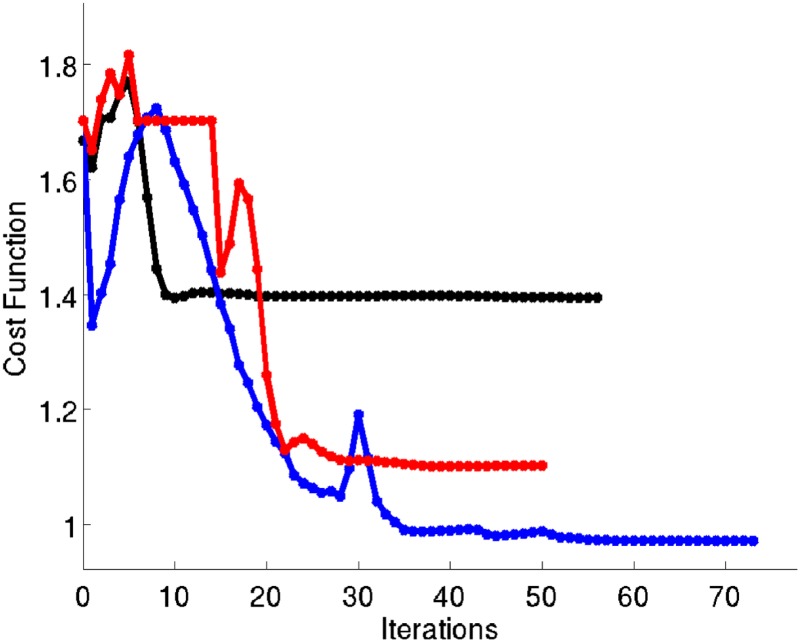
Compliance values of the cantilever beam example along the iterations. In black, the compliance when using the macrostructure topology optimization approach. In blue, the compliance when using the P2P multiscale topology optimization approach. In red, the compliance when using the CB multiscale topology optimization approach

From the performance point of view, in Figure 19, we show the compliance computed by the three approaches. After decreasing a 40% of the mass in the three cases, allowing the possibility of designing the microscopic topology, the structure increases its stiffness in a 30% in the *P2P multiscale topology optimization* problem and in a 21% in the *CB multiscale topology optimization* problem with respect to the *macrostructure topology optimization* solution.

### 4.4 Comments and limitations

In view of the *P2P* and *CB multiscale topology optimization* results, an important improvement of the stiffness (30% and 21%) has been achieved. In addition, the idea of generating the Computational Vademecum offers an appropriate and reduced time‐consuming approach for solving the nonlinear and large‐scale optimization problem. However, there are also some limitations in using the proposed approach. Firstly, the Computational Vademecum is limited to a specific fraction volume. Future should be devoted to explore how to efficiently construct a Vademecum with an extra variable.

Secondly, in the void part of the domain, the microstructure topologies are also optimized. From the physical point of view, this option seems to be unreasonable. However, it is worth stressing that, strictly speaking, the void part behaves as a weak material and designing the microstructure makes perfect sense. To avoid these computations, one may deactivate the void elements.

The scope of this study has been restricted to two‐dimensional problems. The extension to three‐dimensional problems would entail no difficulty, in principle, from the theoretical standpoint. However, from the practical point of view, the computational burden associated to the generation of the Vademecum will increase dramatically, for the parametric space could no longer be represented by the unit sphere. As a remedy, one may replace the *multiscale topology optimization*by a suboptimal problem in which the optimal microstructure topologies are sought in a reduced and representative subdomain of the parametric domain. This study is left for future work.

From the analysis and the results shown in this section, one firstly can observe that the P2P approach really provide an optimal solution. However there is no possibility in making it real since the microstructure changes P2P. Secondly, with the CB approach, one can get a considerable improvement of the cost function fulfilling manufacturable constraints.

In addition, the presented algorithm behaves very efficient in computational terms. This is to be attributed, on the one hand, to the alternate direction algorithm, which provides the solution in relatively few iterations in comparison with other approaches. On the other hand, the idea of precomputing the microstructure optimization problem in a Computational Vademecum has afforded huge computational savings, since it allows the alternate direction algorithm to proceed without solving a microstructure problem.

## 5 Conclusions

This work addressed multiscale topology optimization problems. Accounting for a two‐scale computational homogenization scheme (*F* *E* ^2^), the optimization problem is governed by the influence of the design variables (defined at the microscale or, additionally, at the macroscale) in the cost function (defined at the macroscale). To this aim, we first presented a brief review of what we call the *P2P material design approach* as an alternative to the *macroscopic topology optimization* problem to maximize the stiffness of a structure. Then, in order to achieve manufacturing (and consequently suboptimal) designs, a *CB material design approach* was presented.

To mitigate the unaffordable time‐consuming computations of the problem, a material catalog, which we have termed *Computational Vademecum*, was built as an offline computation. It allows circumventing the microstructure topology design effort in each component by selecting from the *Computational Vademecum* the precomputed optimal microstructure topology. To deal with the strong nonlinearity of the problem, an *alternate direction* algorithm was proposed.

A key ingredient of the *CB material design* approaches is that a considerable improvement of the structural stiffness (15%) was achieved and a few number of iterations are needed to converge the problem. This finding suggests its use in multiscale topology optimization problems. Additionally, we examined the convergence of the *CB material design* approach to the *P2P material design* approach proposed in previous works, which results in a consistent relation between both approaches. Furthermore, the presented efficiency parameter helped in determining the appropriate number of components, which is governed by the tradeoff between the stiffness of the structure and the manufacturability constraints.

A second and even more stimulating part of the study consists in using complementary (instead of alternatively) the *P2P* or *CB multiscale topology optimization* jointly with the macroscopic topology optimization problem. The *P2P* and *CB multiscale topology optimization* provided an additional (around 25% and 15%) increase of the stiffness over the already increase obtained by the macroscopic topology design. In addition, due to the *Computational Vademecum* concept, the problem was solved in less than 10 minutes of computation with a standard PC (3.40 GHz processor in a 64‐bit architecture) in a MATLAB^©^ environment. When examining the results, a strong coupling between the macroscale and microscale, not only from the mechanical point of view but also from the topological point of view, was observed. With the proposed approach, two‐scale topology optimization problems can be solved in a reasonable computational time.

In addition, nowadays, the *Computational Vademecum* concept in tandem with the *CB* approach could be straightforwardly adapted to industrial problems. The current RVEs would be replaced by a standard composite material and the microstructure design variables by the orientation of fibers and number of plies. In the short term, if new optimization tools appear for obtaining improved microscopic topologies, the *Computational Vademecum* can be enhanced by replacing the current microstructure topologies by the improved ones. The microstructures would be replaced, but the *Computational Vademecum* would remain useful. Certainly, additional research is needed to extend the methodology to three‐dimensional problems.
